# Experimental Compaction of a High-Silica Sand in Quasi-Static Conditions

**DOI:** 10.3390/ma16010028

**Published:** 2022-12-21

**Authors:** Krzysztof Szwajka, Marek Szewczyk, Tomasz Trzepieciński

**Affiliations:** 1 Department of Integrated Design and Tribology Systems, Faculty of Mechanics and Technology, Rzeszow University of Technology, Ul. Kwiatkowskiego 4, 37-450 Stalowa Wola, Poland; 2 Department of Manufacturing Processes and Production Engineering, Faculty of Mechanical Engineering and Aeronautics, Rzeszow University of Technology, Al. Powst. Warszawy 8, 35-959 Rzeszów, Poland

**Keywords:** grain size, high-silica sand, loose materials, porosity, strain

## Abstract

In the compaction process, an uneven densification of the powder through the entire height of the die is a major problem which determines the strength properties of the final product, which vary throughout the entire volume. The aim of this investigation was to determine the distribution of the forming pressure inside the die and to visualise the differences in compaction. To determine the pressure inside the die during the compaction process, the deformation on the die surface was measured by means of strain gauges. However, in order to visualise the densification of high-silica sand during the compaction process, an X-ray tomograph was used, which permits one to visualise the interior of the die. The authors developed an analytical model of how the change in internal pressure influences the change in stresses arising on the outer surface of the die, and, as a result, the friction force. It has been observed that the highest values of pressure as well as the highest concentrations of the loose medium are found closest to the punch and decrease with distance from the punch. Moreover, based on the measurements of deformation, a dependence of the pressure distribution on the value of friction forces was observed, which prompted further analysis of this phenomenon. As a result, tests to determine the coefficient of friction between the die and the loose medium were carried out. This made it possible to describe the pressure distribution inside the die, based on the pressure applied and the height of the die.

## 1. Introduction

Currently, processes for the densification of loose media are used in many industries, examples being the foundry industry, in which sand moulds are used, and the pharmaceutical tabletting industry [1,2]. Another example is powder metallurgy [3,4], in which the use of such processes makes it possible to produce elements of machine parts, structural materials or the blades of cutting tools. Manufacturers using this production method struggle with a number of problems linked to obtaining a finished product that has adequate strength while maintaining minimum wear of the mould surface due to friction and high pressures. Granular materials also include granular soils, filling materials in earth-rock structures [5], and wood materials for the production of pellets [6]. The physical properties of loose materials influence the correlation between the particle size distribution and the physical and mechanical properties of the products subject to compaction.

The powder particles are generally irregular in shape. For this reason, their size, given by one parameter, is arbitrary and comes down to determining the equivalent diameter, which, depending on the test method used, is called the sieve diameter when sieve analysis is performed, Feret-Martins diameter—when microscopic analysis is undertaken—and Stokes diameter—when using sedimentation analysis. The most common method of determining the size of powder particles is sieve analysis, which allows the powder to be divided into fractions using a system of sieves arranged one above the other in increasing mesh size. Microscopic analysis consists of comparing the image obtained from a microscope of standard size. Sedimentation analysis uses the phenomenon of powder particles in a liquid having different falling velocities. After establishing the equilibrium state between the force of gravity and the buoyancy force, the particle descends with a uniform motion [7].

As mentioned before, the most significant problem resulting from loose compaction of media is the heterogeneity of the densification of the final product material. This is related to movement both between the particles of the compacted material and at the surface of the die. As a result, the compaction process, apart from the obvious interaction forces between particles, is accompanied by frictional forces between the particles, the bottom of the punch and the walls of the die [8,9]. Because the distribution of the mentioned forces is non-uniform over the entire area of the die, the value of the forming pressure also changes throughout the volume of the material.

In order to learn more about the pressure distribution inside the die, one of three popular methods can be used. These indirect methods make it possible to determine the pressure distribution inside the die on the basis of the deformation of the outer surface of the mould wall. The first and at the same time the most frequently used measurement method is the measurement of deformation using strain gauges [10]. In this method, the main role is played by strain gauges, which, after gluing to the surface of the element being tested and connecting them to the control unit, permit the measurement and registration of deformation on the surface. This method is often used due to its great accuracy and low costs. The advantages of this method include the ability to measure deformation using more than one strain gauge. The second, quite frequently used method of deformation measurement is the method that uses piezoelectric sensors [11,12]. Measurement with this method, as with measurement using the strain gauge method, requires direct contact of the sensor with the surface of the test object. The piezoelectric sensors are mounted on drilled holes. The advantages of this method include the fact that the piezoelectric sensor, unlike strain gauges, is reusable. Another method used to measure deformation is the non-contact system for 3D deformation analysis and digital image correlation (DIC) which consists in overlaying photos taken at different angles with the help of a camera set, resulting in the creation of a graphical map of deformations [13,14]. This method allows the value of the deformation on the entire surface of the test element to be determined.

There have been many investigations of the compaction process of granular materials. Analyses of the compaction process of high-silica sand used in the foundry engineering are very limited. Lowe and Greenaway [9] investigated the role of particle size and porosity on dynamic compaction processes in granular beds in steady state conditions. It was found that for porosity 0.75 ≤ φs ≤ 0.88, the articulate structure of microfine material provides more than three times greater resistance to compaction than coarse bed. Shooshpasha et al. [15] analysed the influence of micro silica content on the compaction properties of cement sands. The results showed that micro silica can be effective in filling the pores of sand-cement mixtures, which can lead to a cement-impregnated sand mixture with a dense microstructure. Humphres [16] developed a method for determining the appropriate maximum density used to control the compaction of granular materials, which eliminates the inhomogeneities often found in the methods currently used. Chester et al. [17] found that the fracturing intensity and degree of fragmentation of aggregates of St. Peter sand increase with volumetric strain, indicating that coarse cracking and grain rearrangement are the main mechanisms of creep compaction. The paper [18] discussed the influence of the coefficient of friction (COF) between particles on the macroscopic and microstructural behaviour of granular materials made of neo-Hookean particles investigated in a quasi-static regime. It was found that in the case of large filling fractions, the strain energy stored in the particles is much greater than the energy dissipated by friction. The stabilising effect of friction reduces the occurrence of elementary events and thus reduces energy dissipation. Mechanisms such as grain rearrangement in combination with grain fracture and elastic deformation play an important role in time-independent compaction of sands. The study of grain size and the influence of loading history, initial porosity and the chemical environment on sand compaction during uniaxial pressing at room temperature was the subject of research by Brzeskovsky et al. [19]. Combining modelling results and experimental tests, it was inferred that a grain-size-dependent departure from sphericity of the grains exerts a key control on the compaction behaviour of sands.

In order to model soft granular systems, many approaches were developed, i.e., the Discrete Element Method (DEM) [18], implicit Material Point Method (MPM) coupled with the Contact Dynamics (CD) method [20,21,22] and methods that consist in combining the Finite Element Method (FEM) with DEM [23,24]. Ransing et al. [25] developed an approach called multi-particle finite element method (MPFEM). Procopio and Zavaliangos [26] found necessary degrees of freedom that allow for local non-uniform contact deformation, which was not available in DEM. Güner et al. [27] carried out numerical modelling of cold powder compaction of multi particle media. They found that friction has a significant effect between the particles and the interaction between the particles and the die surface. The interaction between particles and the die surface causes greater forces of contact friction than interactions between particles. The friction change is related to the shapes of the deformed particles, which results in a more heterogeneous stress distribution along the height of the die.

The method of forming loose materials is a increasingly used in mass production. It is used inthe foundry industry, among other industries, for making foundry moulds, as well as in powder metallurgy. The most significant obstacle that limits the use of this compaction method is the phenomenon of uneven densification of the powder along the entire height of the die, which in turn results in the achievement of a final product whose strength properties change throughout the entire volume. In order to enable a wider use of this method, the undesirable phenomena related to uneven compaction should be eliminated. A problem affecting the frequency of use of loose mass compaction is the rapid wear of dies and punches. This wear is influenced indirectly by the high pressure accompanying the compaction process, but the main parameter influencing accelerated wear is friction. Knowing that the value of the COF changes with pressure change, its characteristics should be determined in order to achieve a correct understanding of the friction phenomena occurring in the die during the pressing process. In this paper, the distribution of forming pressure inside the die during the compaction of high-silica sand has been analysed. The deformation on the die surface was measured by means of strain gauges. In order to visualise the densification of high-silica sand during the compaction process, X-ray tomography was used.

## 2. Materials and Methods

### 2.1. Material

**Figure 1:**
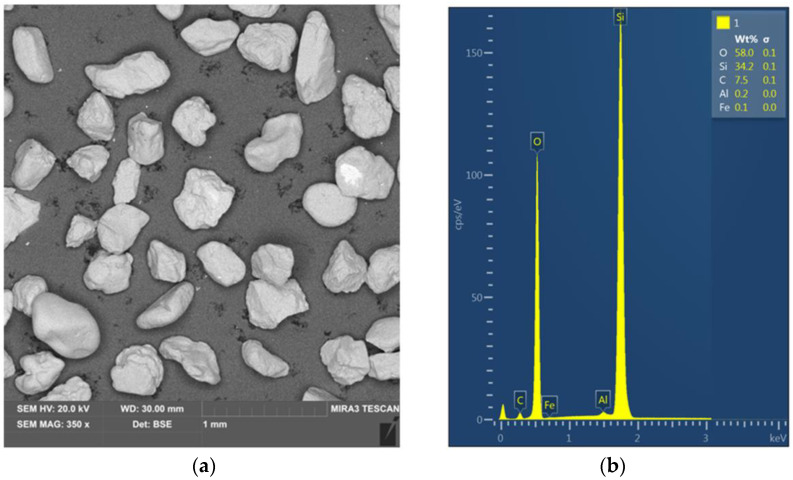
(**a**) SEM micrograph and (**b**) SEM-EDS analysis of the high-silica sand.

The loose material used for the tests was high-silica sand used for the production of foundry moulds. Quartz sand mainly consists of quartz arenite, which is a clastic sedimentary rock with a grain size ranging from 0.0625 mm to 2 mm. The quartz sand was manufactured by Grudzień Las sp. z o.o. (Grudzeń Las, Poland). High-silica sand contains a high proportion of silica (over 95%). The medium was sieved, thanks to which a fraction with similar-sized individual sand grains ranging from 0.4 to 0.6 mm was obtained. In order to visualise the grain size of the medium prepared for testing, a sample was prepared and examined using a scanning electron microscope (TESCAN, MIRA3, Brno, Czech Republic). SEM micrograph and an SEM-EDS spectrum of the high-silica sand are presented in Figure 1a,b, respectively.

**Figure 2:**
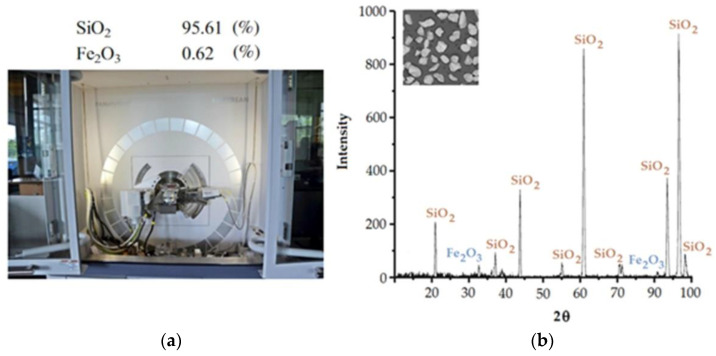
(**a**) X-ray diffractometer and (**b**) XRD spectrum of silica sand.

X-ray diffraction (XRD) analysis was performed on a diffractometer Empyrean (Figure 2a) (Malvern Panalytical Ltd., Malvern, UK) The main phases found in the test sand sample are composed of silica SiO_2_ (about 95.61%) and iron oxide Fe_2_O_3_ (about 0.62%). The results of the XRD analysis of the samples are shown in Figure 2b. A large fluctuation of the SiO_2_ content can be observed for the 2Q angle. The highest peaks in the XRD analysis presented indicate the presence of SiO_2_ as the main crystal of the mineral, which is very advantageous as it provides the permeability, fire resistance and chemical resistivity of the casting mould model. Other minerals scattered in the sand are Fe_2_O_3_, which have very small peaks (Figure 2b). Their content in the sand is 0.62%, which is a small percentage compared to the content of SiO_2_.

### 2.2. Compaction Setup

**Figure 3:**
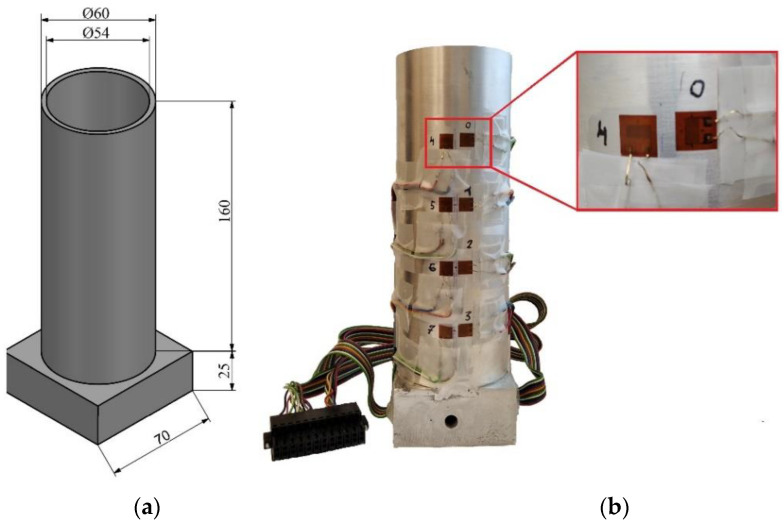
(**a**) dimensions (in mm) of the cylindrical die used in the experiments and (**b**) method of attaching strain gauges to the die surface.

To analyse the pressure distribution inside the die during the compaction process, a cylindrical die was used. The geometrical dimensions of the die prepared for testing and the method of sticking strain gauges onto the die surface are shown in Figure 3a,b, respectively. The die and base were made of EN AW-2017 aluminium alloy, which has good tensile and fatigue properties. Both the tube and the base were welded together. Selected physical properties of EN AW-2017 alloy are presented in Table 1.

In the investigations on the measurement of the distribution of stresses during the pressing process, it was decided to carry out measurements using strain gauges. The selection of this method of measurement was dictated by the fact that it is an accurate and cheap method of determining stresses on the basis of strains. The measurement of the deformation on the surface of the die was carried out with the use of strain gauges, the arrangement of which was selected in order to observe the change in strain values through the entire height of the die. Additionally, in order to better understand the distribution of stresses both in the axial and tangential directions, two strain gauges arranged perpendicularly to each other were glued to the surface of the die, as shown in Figure 3b.

**Figure 4:**
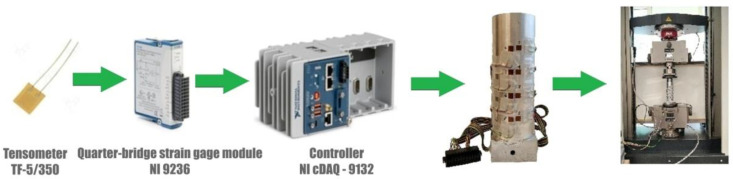
Diagram of the measurement track.

**Figure 5:**
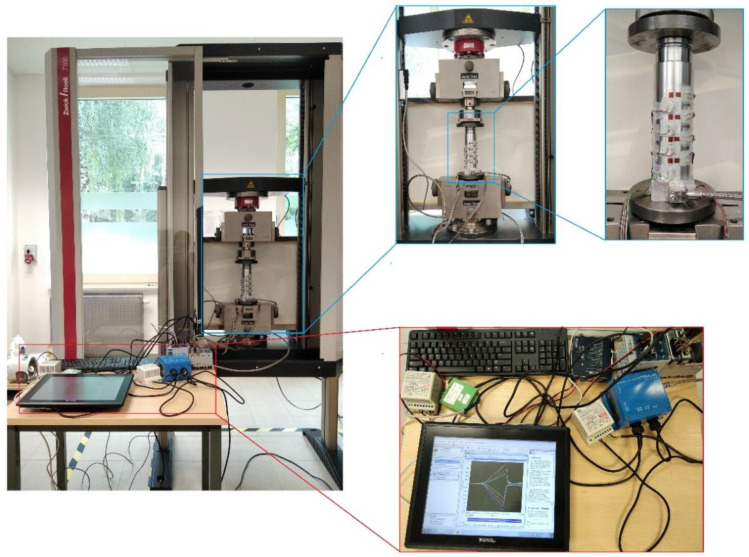
Strain measurement stand.

The measurement of deformation on the die surface during the process of densification of the loose medium was carried out using the National Instruments stand, consisting of the NI cDAQ-9132 controller and the NI 9236 deformation measurement module. The strain gauges glued on the die surface were connected to this module (Figure 4). The parameters of the TF-5/350 foil strain gauges are listed in Table 2. Measurements of die deformation during quasi-static compaction of the loose medium were carried out on a measuring stand mounted on a Zwick/Roell Z100 testing machine (Figure 5). A die (Figure 3) was mounted in the lower fixture of the testing machine, while the punch was mounted in the upper fixture. During the experiment, the force and displacement of the punch were recorded at a frequency of 5 Hz. The displacement speed of the punch was 10 mm/min. The high-silica sand inside the die was loaded with a force of 80150 N with an increment of 2290 N.

**Figure 6:**
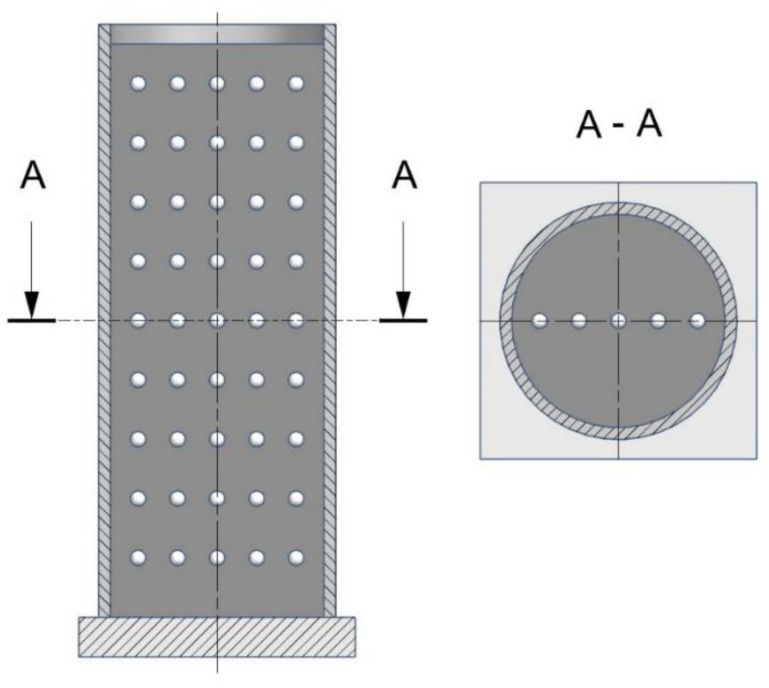
Diagram of the arrangement of the balls in the die.

Visualisation of the change in densification was carried out using an X-ray tomograph, which allowed the observation of the behaviour of the loose medium inside the die based on the displacement of the points, which were layers of lead balls with a diameter of 4 mm, as shown in Figure 6. The distance between the layers of the balls was approximately 15 mm. The charge was compressed to a pressure of 15 MPa.

**Figure 7:**
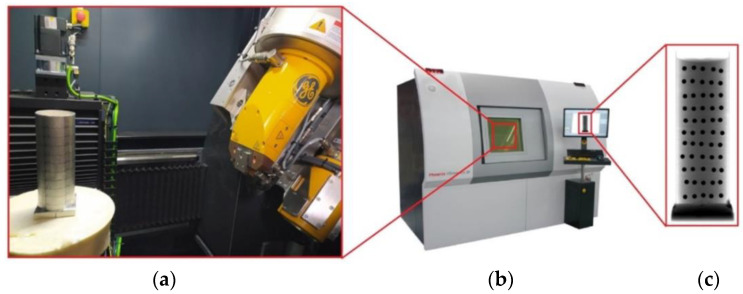
X-ray test stand, (**a**) setting the die on the rotating X-ray tomograph, (**b**) General Electric phoenix v|tome|x m X-ray tomograph, (**c**) X-rayed die.

The die shown in Figure 7 was X-rayed and photographed using the General Electric phoenix v|tome|x m X-ray tomograph. In order to correctly estimate the densification inside the die stamping as a result of pressing, it was necessary to know the value of the displacements of the individual layers of the balls. Therefore, the X-ray process was carried out twice, i.e., before and immediately after the compaction process.

### 2.3. Surface Roughness Measurement

**Figure 8:**
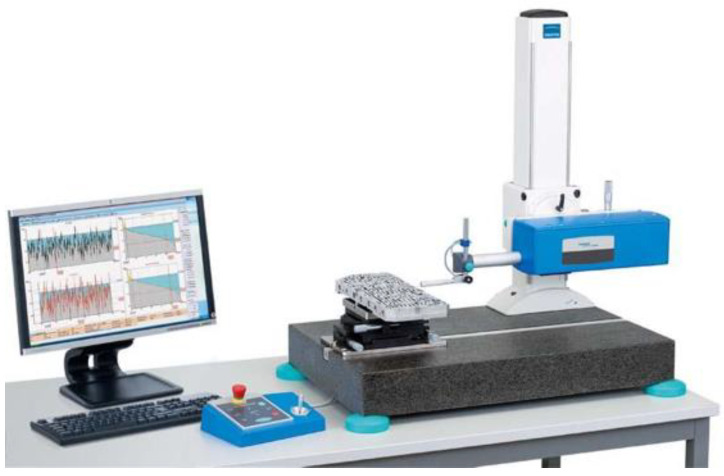
Stand of surface topography measurement.

**Figure 9:**
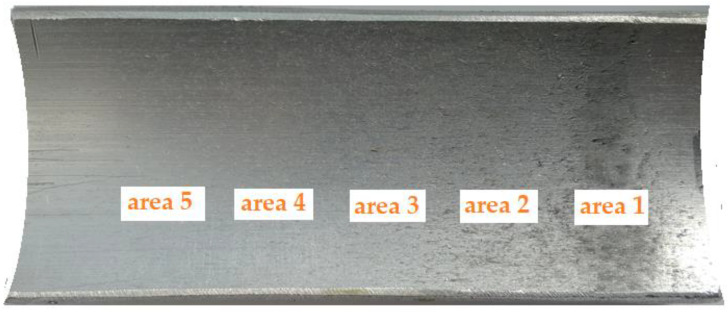
Areas in the die used for the measurement of surface topography.

For selected areas of the die, the surface topography obtained was measured using a Hommel-Etamic T8000RC CNC profilometer (Jenoptik, Jena, Germany) (Figure 8). Figure 9 shows a photograph of the die section that was used in the research. Surface roughness was measured in five areas 3 mm long and 3 mm wide.

### 2.4. Sieve Analysis

**Figure 10:**
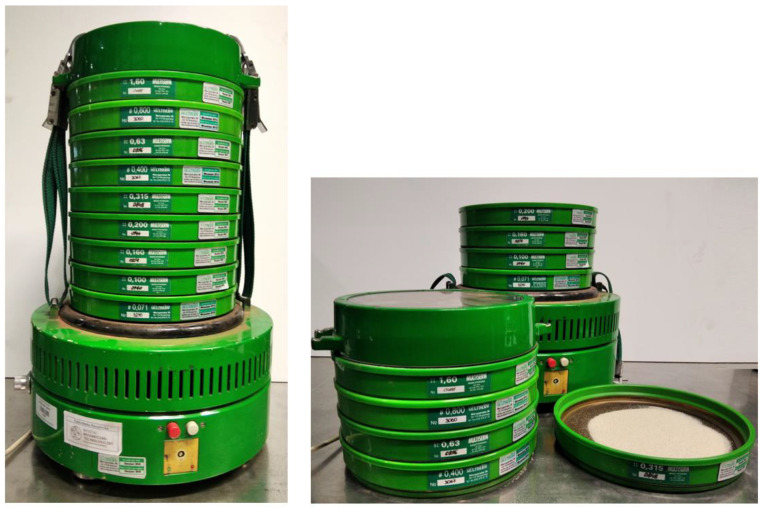
Sieve shakerLPzE-2 with a set of sieves 0.071–1.6 mm.

The sieve analysis consists in determining the granulometric composition of the sand by separating individual sand fractions as a result of sieving the sample through a set of standardized sieves. Sieve shaker LPzE-2 (Multiserw-Morek, Brzeźnica, Poland) (Figure 10) equipped with a steel sieve frames with a wire mesh sieve was used for the tests. The set of sieves used included sieves with a mesh diameter from 1.6 mm to 0.071 mm and a collection vessel. The number of sieves used allowed to ensure the continuity of the grain size curve. The fraction with the grain size between 0.315 mm and 0.4 mm (fifth sieve) was used for compaction process.

## 3. Results

### 3.1. Coefficient of Friction

**Figure 11:**
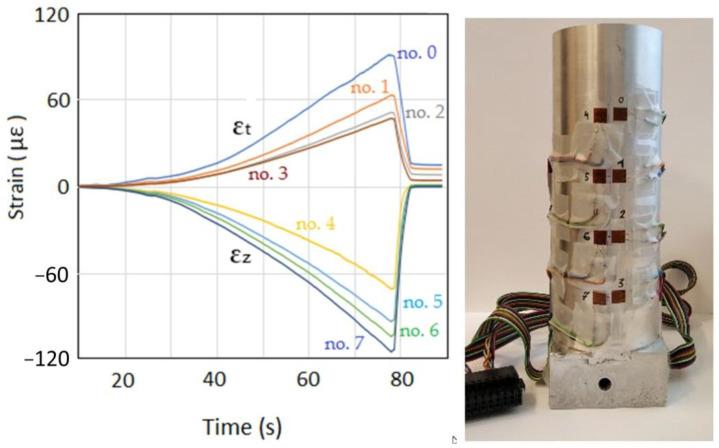
The distribution of strains with height in the die during the compaction of high-silica sand in the axial (ε_z_) and tangential (ε_t_) direction.

The values of the strains of the die surface during the compaction of sand that were recorded are shown in Figure 11. The largest tangential strains (ε_t_) were recorded near the surface of the punch (no. 0) and the values of the tangential strains of the die walls decreased exponentially with distance from the punch surface. The situation was different for the axial strains (ε_z_). The highest value of axial strain was achieved in the place furthest from the punch surface (no. 7). This is understandable because the external friction forces are “summed up” as the measurement site moves away from the punch surface. As in the case of the tangential strains of the die walls, the changes in the values of axial strains were also exponential.

**Figure 12:**
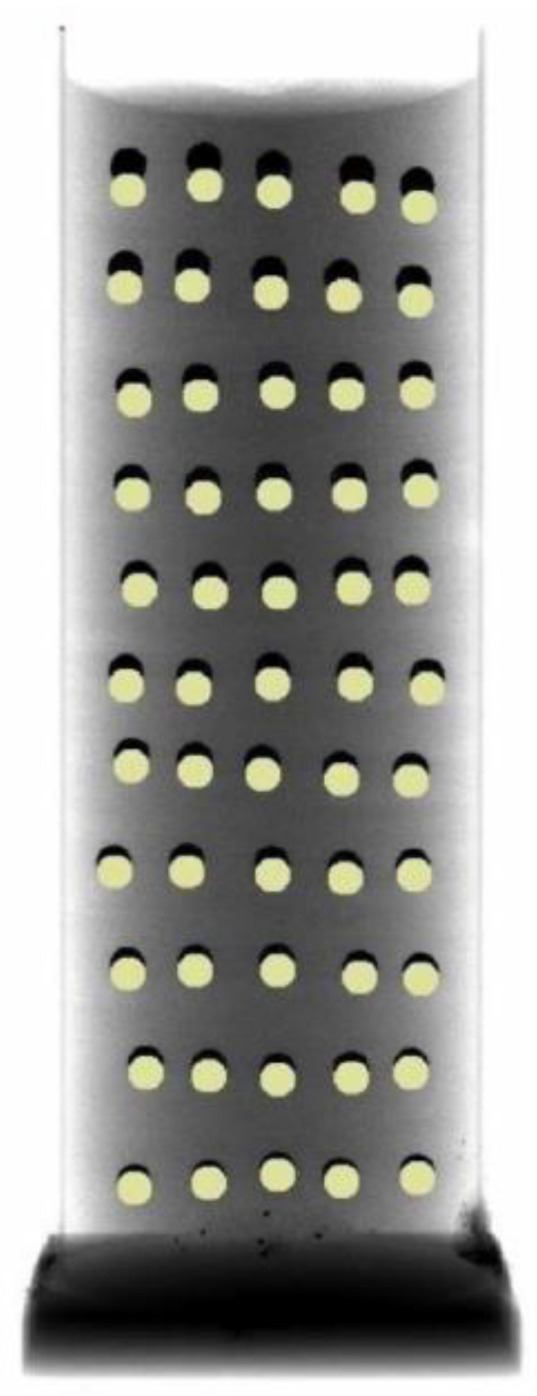
An X-ray tomography scan of the position of the balls before (black outline) and after (yellow outline) the compaction process.

**Figure 13:**
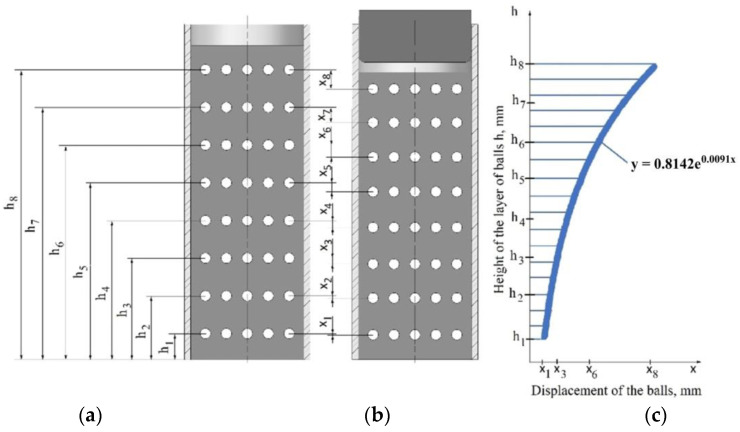
Distribution of lead balls (**a**) before and (**b**) after the compaction process, and (**c**) displacement vs. die height function of the balls displacement vs. die height.

During these tests, it was found that the value of the pressure inside the die changes as a function of the distance from the punch surface. This hypothesis was confirmed by the results of the research, in which the changes in the density of the loose medium during the compaction process were analysed. Figure 12 shows the superimposed position of the balls before the forming process (black outline) and after the forming process (yellow outline). The changes that were registered in the position of the balls indicate a different character of powder compaction with height in the die. The diagram quantifying the displacement of the balls after the pressing process is shown in Figure 13.

The dependence of the changing value of ball displacement on distance from the punch surface can be described by an exponential function. The greatest displacement of the balls was observed closest to the surface of the punch. The distance of ball movement decreased with increasing the distance from the punch surface. This clearly determines the variable character of the compaction of the loose material inside the die and thus confirms the variable nature of the pressure distribution inside the die during the compaction process.

The pressure distribution in the die is dependent on both the particle size and the shape of the silica sand. The contact surface of grains after loose backfilling is very small. During compaction of the powder, the powder grains are brought closer together, which enables the formation of adhesion forces and the enlargement of the contact surface of the grains by crushing and bringing them together. The porosity of the powder depends on the shape and size of the powder particles and its granularity. Powders with a more irregular shape and developed surface are characterized by a large contact surface. Displacements of powder particles with low roughness are smaller, so the pressure distribution is more even across the cross-section of the compact.

Due to the different value of the coefficient of friction between the powder particles and between the powder particles and the die wall, as well as the facilitated movement of the particles in the central zone of the compact, obtaining a uniform deformation field is difficult. The uniformity of deformation field can be increased by applying protective coating on the internal surface of the die, which reduces the value of the coefficient of friction.

**Figure 14:**
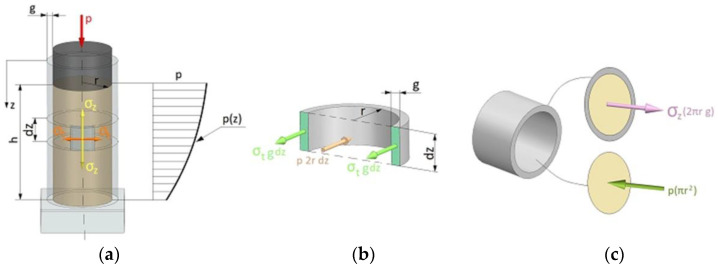
(**a**) pressure distribution p(z), (**b**) circumferential stresses, (**c**) longitudinal stresses in the compaction process.

To calculate the pressure acting on the walls of the die, the hypothesis of a homogeneous medium was adopted, to which the equations of continuum mechanics can be applied. It was assumed that the pressure p(z) at a given height of the sample and throughout its cross-section is constant in value (Figure 14c). The diagram of the moulding sand compaction process and the circumferential stress distribution are shown in Figure 14a,b, respectively. In the analysis, the function of pressure distribution inside the die p(z) was determined using computed tomography. Knowledge of the distribution of internal pressure will allow one to determine the value of the pressure force N(z) (Figure 13). As can be seen, the maximum internal pressure p(z) occurs right next to the punch surface and is close to the value of the set compaction pressure p. Such distribution of internal pressure p(z) is caused by friction forces. The total friction force causes the internal pressure p(z) to decrease with the distance from the punch surface. This is due to the increase in the coefficient of external friction µ(z) with the decrease in the value of internal pressure p(z). To sum up, the computed tomography analysis allowed only to determine the change in the pressing pressure inside the die and, as a result, the change in the value of the pressing force N(z) as a function of the distance from the punch surface.

**Figure 15:**
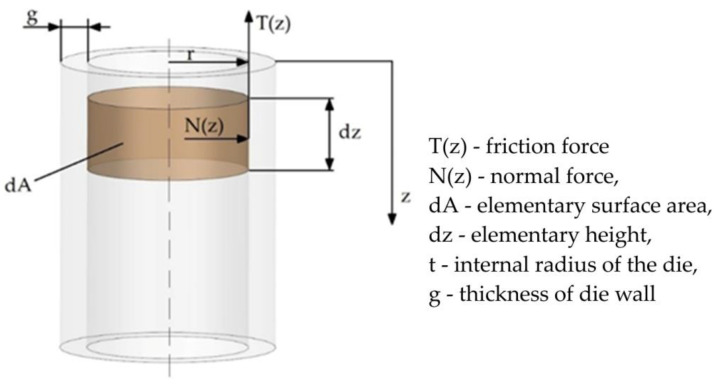
Calculation diagram for the determination of the external coefficient of friction.

The force with which two contacting bodies interact and oppose their relative motion is called friction force T. The friction force has a direction tangential to the contact surface, acts on each body, and has a sense opposite to that of its velocity (Figure 15). The source of the friction force lies in the interactions between the molecules of the bodies in contact. If the bodies do not slide against each other, the resulting frictional force is called the static friction force. If the bodies move relative to each other, the forces of kinetic friction act between their surfaces.

In this study, we have a case of kinetic friction. The friction force does not depend on the size of the contact area and is proportional to the normal reaction force. The ratio of the maximum friction force T and the reaction force N is called the coefficient of friction:

(1) $$µ=\frac{T}{N}$$

**Figure 16:**
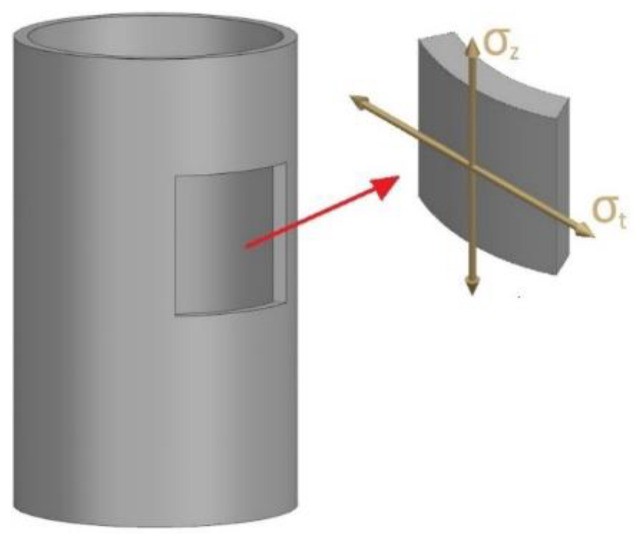
Location of hoop and axial stresses in the die wall.

To determine the friction force between the die wall and the medium, the values of hoop stress ($\sigma_{t}$) were used (Figure 16).

**Figure 17:**
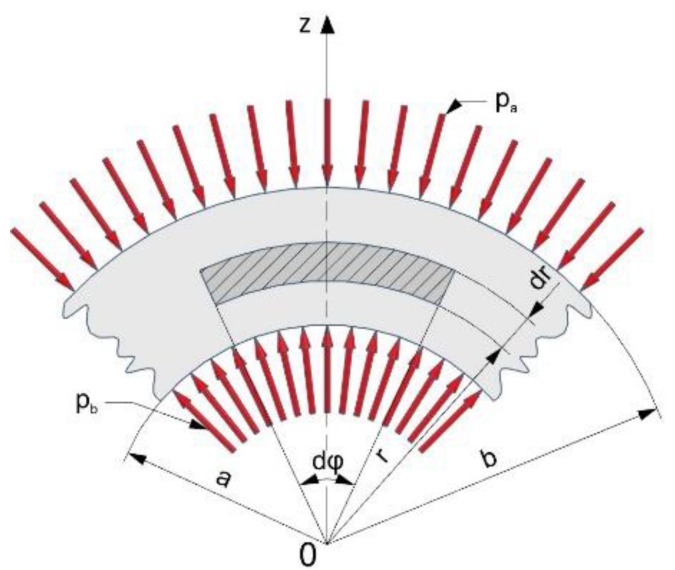
Loads acting on the elementary fragment of the die wall.

The value of the hoop and axial stresses are usually determined from the Lame formulae, which have the form:

(2) $$\sigma_{r}=\frac{p_{a}a^{2}}{b^{2}-a^{2}}\left(1-\frac{b^{2}}{r^{2}}\right)-\frac{p_{b}b^{2}}{b^{2}-a^{2}}\left(1-\frac{a^{2}}{r^{2}}\right)$$

(3) $$\sigma_{t}=\frac{p_{a}a^{2}}{b^{2}-a^{2}}\left(1+\frac{b^{2}}{r^{2}}\right)-\frac{p_{b}b^{2}}{b^{2}-a^{2}}\left(1+\frac{a^{2}}{r^{2}}\right)$$

where the geometrical parameters and loading of the elementary fragment of the die wall are explained in Figure 17.

The above formulae for calculating the stress values in the pipe surface apply only to pipes with constant internal pressure. In the present case, the values of the internal pressure are variable. The authors attempted to determine how the change of internal pressure influences the change in the stresses arising on the outer surface of the die, and, as a result, the friction force. The following analysis was performed under conditions where a variable internal pressure is exerted on the die.

Assuming a variable value of the friction force T(z) as a function of the die height, the following relation can be written:

(4) T(z)= μ(z)·N(z)

In Equation (4), both the variable value of the coefficient of friction μ(z) and the normal force N(z) were assumed, which depend on the prevailing internal pressure.

Taking into account that the normal force N(z) can be expressed as the product of the internal pressure p(z) and the elementary surface area dA, we get:

(5) dT=p(z)· dA · μ(z)

Substituting in the elementary surface area dA, the following expression:

(6) dA=2πr · dz

where dz is the elementary height increment of the die, we get the elementary value of the friction force dT:

(7) dT=p(z)· μ(z)·2πr ·dz

By integrating the Equation (7) we obtain the friction force in the form:

(8) $$T\left(z\right)=2\pi r\;\overset{z}{\underset{0}{\int }}p\left(z\right)\mathrm{dz}\;\cdot \;\mu \left(z\right)$$

As can be seen in Equation (8), the friction force depends both on the value of the internal pressure and the coefficient of friction. The value of axial stress σ_z_ can be written in the form (9):

(9) $$\sigma_{z}=\frac{T\left(z\right)}{2\pi \mathrm{rg}}$$

The elementary value of the axial stress dσ_z_ can be expressed as:

(10) $${d\sigma }_{z}=\frac{\mathrm{dT}}{2\pi \mathrm{rg}}$$

Substituting Equation (8) into Equation (10) we get:

(11) $${d\sigma }_{z}=\frac{\mu \left(z\right)\cdot p\left(z\right)\cdot 2\pi r\;\cdot \;\mathrm{dz}}{2\pi \mathrm{rg}}$$

and after simplification:

(12) $$\frac{{d\sigma }_{z}}{\mathrm{dz}}=\frac{\mu \left(z\right)\cdot \;p\left(z\right)}{g}$$

Finally, the expression for determining the value of the coefficient of friction takes the form (13):

(13) $$\mu \left(z\right)=\frac{{d\sigma }_{z}}{\mathrm{dz}}\cdot \frac{g}{p\left(z\right)}$$

**Figure 18:**
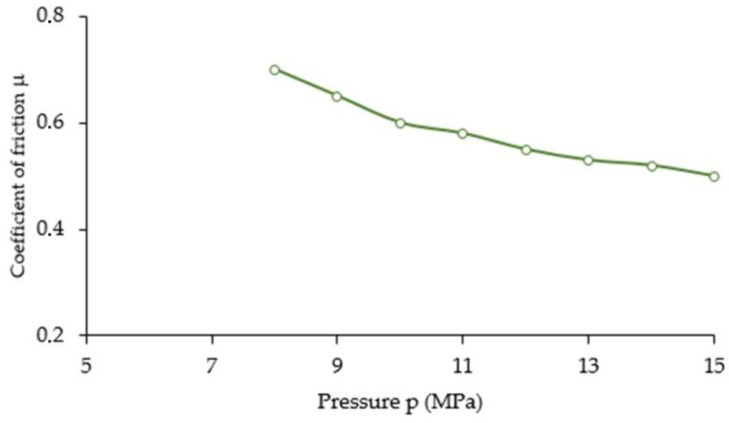
The effect of punch pressure p(z) on the coefficient of external friction (μ).

Using Equation (13), it is possible to indirectly determine the value of the coefficient of external friction µ(z) depending on the distance from the punch surface. The value of the coefficient of friction has been determined based on the form of the function σ_z_(z) describing the stress distribution on the outer surface of the die, which was obtained in experimental measurements carried out using resistive strainometry, and the pressure distribution function p(z), which was obtained by analysing the images from X-ray tomography. Figure 18 shows the values that were obtained for the coefficient of external friction as a function of pressure. As can be seen, in the case analysed, the value of the coefficient of friction µ(z) decreases with increasing pressure. Since local high pressures occur during compaction, the contact stresses may exceed the yield stress of the compacted material or the ultimate cracking stress. In this case, the solid particles cannot be considered non-deformable: even if the initial particle shapes are regular, these shapes are constantly evolving during compaction. The smaller the grains of the compacted mixture, the lower the coefficient of external friction.

### 3.2. Surface Topography

Surface topography is one of the main features taken into account in assessing surface quality in compaction of the loose processes. The value of the Sq parameter for surface roughness was measured in the course of the investigations. The Sq parameter is a root mean square height of the surface. The root mean square height or Sq parameter is defined as the root mean square value of the surface departures z(x,y), within the sampling area [28]:

(14) $$\mathrm{Sq}=\sqrt{\frac{1}{A}\overset{}{\underset{A}{\iint }}z^{2}\left(x,y\right)\mathrm{dxdy}}$$

where: A—area, x, y—lengths in perpendicular directions; z—surface height position x, y.

Sq is a statistical parameter with a relatively low sensitivity to measurement errors. It is often used in surface topography measurements. This parameter is related to the standard deviation of roughness height, which is often used in contact mechanics.

**Figure 19:**
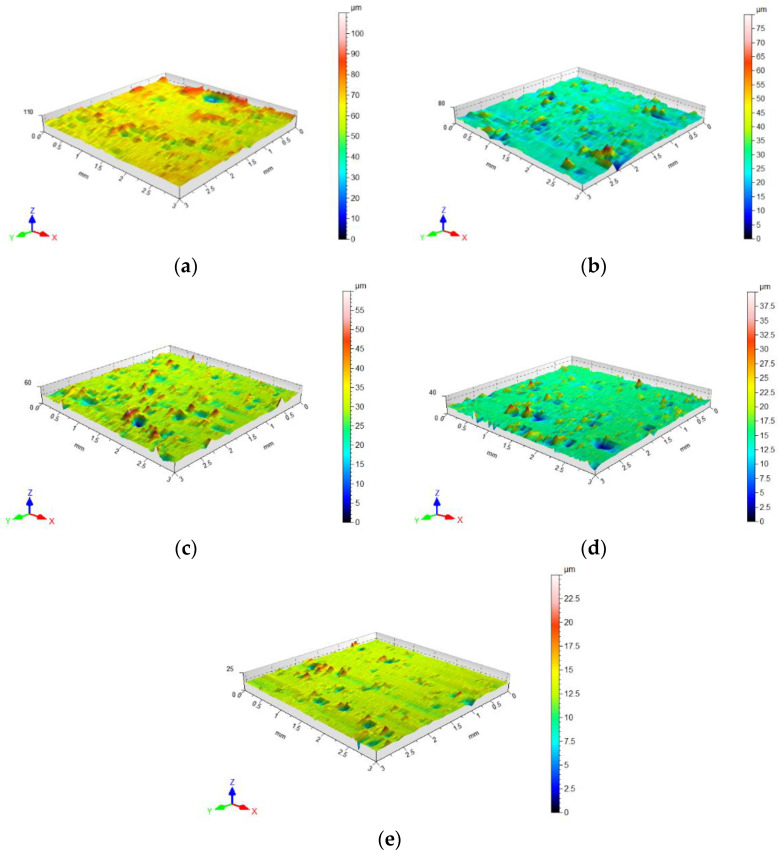
Surface topography in selected areas of the die surface: (**a**) area 1; (**b**) area 2; (**c**) area 3; (**d**) area 4; (**e**) area 5.

Die surface topographies obtained in the compaction process, in the locations shown in Figure 9, are shown in Figure 19. The value of the Sq parameter was determined from the topography maps shown in this figure. On the surface topography maps presented, one can clearly observe that the surface topography changes depending on the height of the die. The first (area 1) and second (area 2) areas appear in the immediate vicinity of the punch surface, where the highest compaction of the high-silica sand was recorded as were, at the same time, the highest tangential stresses.

**Figure 20:**
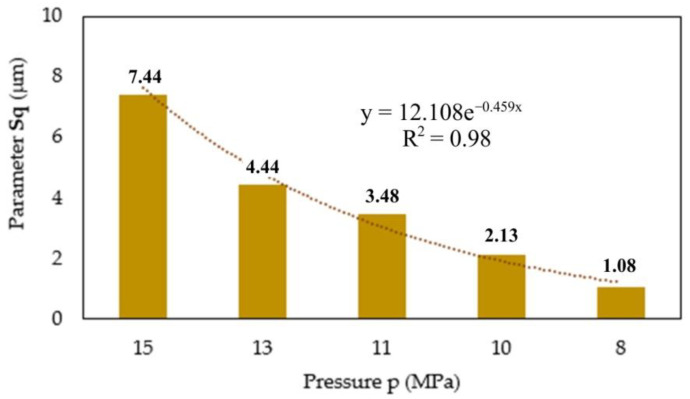
Effect of the pressure on the root mean square height of the surface.

In the analysis of the surface topography, the measurement of the roughness parameter Sq was performed separately for five areas of the die surface. Figure 20 shows the effect of the pressure on the root mean square height of the surface. It can be observed that the change in the value of the Sq parameter is exponential, in a similar manner to the pressure distribution in the die. The largest value of the Sq parameter occurs near the punch surface. Thereafter, this value decreases as it moves away from the surface of the punch.

## 4. Conclusions

In this paper the compaction of high-silica sand used in foundry engineering applications was carried out. Based on the results obtained, the following conclusions can be drawn:

- changes in the value of strains of the external walls of the die (both axial and tangential) are exponential as a function of the distance along the axis of the punch. The greatest tangential strains (ε_t_) were recorded near the surface of the punch. On the other hand, the highest value of axial strain (ε_z_) was achieved in the place farthest from the surface of the punch;
- the compaction of high-silica sand grains along the punch axis can be described by an exponential function. Layers closer to the surface of the punch undergo greater deformation, which results from, among others factors, the lower value of the coefficient of friction between the material to be compacted and the inner wall of the die;
- the tests conducted have shown the usefulness of measuring the deformation on the outer surface of the die for indirect determination of the value of the coefficient of external friction;
- the distribution of the pressure p(z) in the direction of the punch axis during compaction of high-silica sand can be described using X-ray tomography analysis;
- the friction forces cause a reduction in pressure with increasing distance from the punch surface;
- the value of the coefficient of friction µ(z) decreases with increasing pressure p(z).

The study of compaction of high-silica sand of varying density along the height profile of the die will be the subject of a forthcoming study with the aim of analysing die stamping heterogeneity and densification and the resulting spatial heterogeneities for various coefficients of friction.

## Figures and Tables

**Table 1:** Selected physico-mechanical properties of the EN AW-2017 aluminium alloy.

Property	Unit	Value
Hardness HB	-	110
Density	g/cm^3^	2.79
Poisson’s ratio	-	0.33
Coefficient of thermal expansion	°C^−1^	22.9 × 10^−6^
Specific resistance	nWm	51
Young’s modulus	MPa	72,500

**Table 2:** Parameters of the TF-5/350 strain gauge.

Parameter	Unit	Value
Resistance	Ω	350 ± 0.2%
Width	mm	5.0
Length	mm	8.5
Thickness	µm	60
Maximum current, mA	mA	50
Temperature range	°C	−40–200
Fatigue strength	-	n > 10^7^ for ε = 0.1%
Maximum strain	%	approx. 4
Strain sensitivity factor k	-	2.1–2.2
Tolerance of coefficient k, %	%	0.5

## Data Availability

Data are contained within the article.
